# Psychophysics of user acceptance of social cyber-physical systems

**DOI:** 10.3389/frobt.2024.1414853

**Published:** 2024-10-15

**Authors:** Maya Dimitrova, Neda Chehlarova, Anastas Madzharov, Aleksandar Krastev, Ivan Chavdarov

**Affiliations:** ^1^ Institute of Robotics, Bulgarian Academy of Sciences, Sofia, Bulgaria; ^2^ Department of Mathematics and Informatics, Sofia University, Sofia, Bulgaria

**Keywords:** cyber-physical systems, robot acceptance, psychophysics, psychological reactance, categorical perception, uncanny valley effect, probability distribution, accessibility

## Abstract

A mini-review of the literature, supporting the view on the psychophysical origins of some user acceptance effects of cyber-physical systems (CPSs), is presented and discussed in this paper. Psychophysics implies the existence of a lawful functional dependence between some aspect/dimension of the stimulation from the environment, entering the senses of the human, and the psychological effect that is being produced by this stimulation, as reflected in the subjective responses. Several psychophysical models are discussed in this mini-review, aiming to support the view that the observed effects of reactance to a robot or the uncanny valley phenomenon are essentially the same subjective effects of different intensity. Justification is provided that human responses to technologically and socially ambiguous stimuli obey some regularity, which can be considered a lawful dependence in a psychophysical sense. The main conclusion is based on the evidence that psychophysics can provide useful and helpful, as well as parsimonious, design recommendations for scenarios with CPSs for social applications.

## 1 Introduction

The introduction of cyber-physical systems (CPSs) has become ubiquitous, raising issues of adequate design and reliable interdependencies between their different components (technical and social), being in itself of a large societal impact (Losano and Vijayan, 2020; Wang et al., 2023; El-Haouzi et al., 2021). Some of these CPSs are especially designed with interfaces, capable of conveying social communication (such as chat bots in various applications) and education, e.g., LEGO (Lawhead et al., 2002) or MIRO (Collins et al., 2015) robots, as well as performing psycho-social or pedagogical rehabilitation roles (Bachrach, 1992; Dimitrova et al., 2023; Robinson et al., 2019; Dimitrova et al., 2021a). The CPSs in focus in the present mini-review are those used for social applications mostly, as given in the book (Dimitrova and Wagatsuma, 2019). A functional classification of the existing CPSs, consisting of 10 groups of the respective technological devices/systems, is proposed in Dimitrova et al. (2016). Eight of these are being commonly referred to in respect to their functional roles, such as CPSs for manufacturing, agriculture, transport, energy, medicine, and disability. Two more types are added—“CPSs for creativity, art, social communication/media and companionship” and “CPSs for education and pedagogical rehabilitation” (which were further explored within the EU-funded research project “CybSPEED: Cyber-physical systems for pedagogical rehabilitation in special education,” 2017–2023)^1^. It is evident that these two types of CPSs have emerged in the lifetime of the most recent human generations; therefore, investigating their socio–cultural relevance from different scientific perspectives—including psychophysical and psychosocial—is still ahead.

The paper is an attempt to relate the “uncanny valley” effect, as described originally by Masahiro Mori in his seminal work, reproduced in Mori et al. (2012), and some laws in psychophysics, which can explain and predict the intensity of a human reaction to a mechanical device, intended to perform social roles and, therefore, perceived as a special category/ontology of “being,” sharing the features of *sentient*, *living*, and *nonliving* things (Prescott, 2017; Moore, 2012; Dimitrova and Wagatsuma, 2015).

A point of terminological departure in the present mini-review is the attempt to make (as much as possible) a clear distinction between *metaphors* and real *entities/processes/objects,* which are often used in human–robot interaction research. For example, a “social robot” is a metaphor, whereas a “social CPS” is a real entity—a CPS for a social application like a robot used in teaching children social skills. The “uncanny valley” is a metaphor, whereas “psychological reactance” is a real process, which is being measured in the respective scientific discipline (psychometrics). In a similar manner, we refer to “user acceptance” of social CPSs as to a real psychological effect, differing and being measurable in various conditions, which may, or may not, correlate with other psychological effects like, trust, compliance, and liking.

Numerous factors of user acceptance of socially functioning CPSs (Horváth, 2014) have been intensively studied recently within frameworks like the technology acceptance model (TAM) and the unified theory of acceptance and use of technology (UTAUT) and summarized in previous studies (Wolbring et al., 2013; Ghazali et al., 2018; Ghazali et al., 2020). Furthermore, user acceptance of *persuasive* robots is being modeled within the recently proposed persuasive robot acceptance model (PRAM), demonstrating that the user trusting and liking the robot added predictive power to the PRAM. However, psychological *reactance* and compliance, being the processes of interest in the present mini-review, “were not found to contribute to the prediction of persuasive robot’s acceptance” (Ghazali et al., 2020, p. 1,075). This outcome suggests that the perception of the *persuasion* from the robot (an explicit process) is not directly linked to the self-reflection of *reactance* to the robot (an implicit process) (Ghazali et al., 2018). Therefore, employing a methodology beyond psychometrics can be helpful in revealing important aspects, contributing to the user experiencing *comfort* with the CPS and providing other useful guidelines for technology design.

Unlike numerous studies on factor similarity, contributing to user acceptance of robotic systems, our focus is on defining the inner psychological *dynamics* of a human encounter with a CPS in terms of its *intensity* (measured response to a range of stimulations) and its *valence* (type of emotional reaction, i.e., positive or negative). The main assumption in the analysis here is the understanding that the “uncanny valley” and “reactance” to the robot are the same as psychological effects but differ in their emotional depth—the former is deeper and the latter is shallower. In this way, different roles of robots can be allocated depending on users being confident with different styles of interaction with mechanical, machine-looking, humanoid, or android devices.

This mini-review presents some classical psychophysical assumptions, which are relevant to the issue of CPS acceptance as a psychological reality, in Section 2. Within this subjective realm, by applying Thurstone’s model (e.g., the “law of comparative judgement”) (Thurstone, 1927; Thurstone, 1928), the individual distance between the perceived agents on the *human likeness* dimension (x-axis) can be defined and *facets* of the reaction of the human toward the agents (y-axis) assessed, as described in Section 3, which summarizes the main points of the paper, proposing the implementation of indirect scaling procedures to predict user confidence with specific CPSs/robots performing different social roles.

## 2 Classical psychophysical assumptions relevant to user acceptance of social CPSs as a psychological reality

Masahiro Mori defined the “uncanny valley” effect in *psychophysical* terms, writing that “the mathematical term monotonically increasing function^2^ describes a relation in which the function y = f(x) increases continuously with the variable x … I have noticed that, in climbing toward the goal of making robots appear like a human^3^ our affinity for them^4^ increases until we come to a valley … , which I call the uncanny valley” (Mori et al., 2012, p. 98).

The *small* increment of x, bringing the “uncanny valley” emotional response from liking to repulsion with the increase in “human likeness” beyond a certain degree, is something that resembles the so-called “just noticeable difference” (JND) in psychophysics (Sanford and Halberda, 2023). This increment in x is commonly assumed as the quantitative basis for devising the so-called “psychophysics laws” of perception of Weber, Fechner, or Stevens (Laming, 2009; Fechner, 1948; Stevens, 1957). For example, Weber’s law assumes that the JND of a stimulus (Δx) is relative to the intensity of this stimulus x, so under different conditions, the JNDs are different in magnitude (when x is big, Δx is big and *vice versa*, so that Δx/x = constant) (Laming, 2009). Fechner’s law postulates that—in strictly controlled experimental situations—the subjective experience is a logarithmic function of the stimulation (Fechner, 1948). Stevens proposed a power function to express the same relation, where the power can be > or < than 1 (Stevens, 1957). In general, it was found that these *laws* are valid under strictly regulated experimental conditions and with respect to sets of intensities of “pure” stimuli like pitch, illumination, and amounts of a chemical substance for taste. Further studies demonstrated the *complexity* of brain processing, underlying every *sensation* reported by the subject, as is the processing of *abstract concepts* like the “human likeness” (Deco and Rolls, 2006; Johnson et al., 2002).

Of main interest in psychophysics are some boundary conditions when the subject is not entirely sure in her/his assessment in each trial, yet in multiple trials, the threshold of sensitivity to the minimal increase (Δx) in the physical quantity of the stimulation is being determined (the so-called differential threshold) (Sanford and Halberda, 2023). That is, two different, but close, values of a given intensity x_1_ and x_2_ on the stimulus dimension x, where x_2_–x_1_ = Δx, are represented by the means M_1_ and M_2_, respectively, of two different but close distributions of psychological effects, where ΔM = M_2_–M_1_. The psychophysical methodology is often based on using some methods for *indirect* measurements to evaluate the above subjective values, as is the “law of comparative judgement” proposed by Thurstone (1928) or the “law of categorical judgement” proposed by Torgerson (1958). Thurstone’s model is based on a scaling method for *pairwise comparisons* of stimulus intensities x_1_, x_2_, … x_n_, resulting in the respective values of the means of the subjective effects’ distributions M_1_, M_2_, … ,M_n_. The distances between the means are being plotted on an *interval* scale (after computing the probabilities of the relations of the pairwise comparisons and respective z transformations, omitted here for simplicity) (Franceschini, and Maisano, 2020).

**FIGURE 1:**
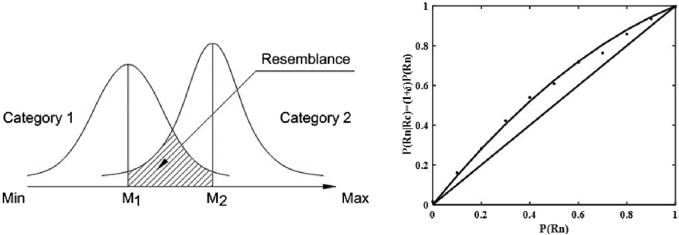
Hypothetical representation of two “nearly independent” distributions of effects produced by the categories 1 (“non-human”) and 2 (“human”) (left). Reproduced from Dimitrova et al. (2021b), licensed under Creative Commons Attribution License (CC BY). A hypothetical plot of Tulving and Wiseman’s “near-independence” function of P(Rn) along the x axis and P(Rn|Rc) = (1+ δ)P(Rn) along the y axis (right).

Our proposal is to use Thurstone’s scaling method in order to arrange a set of robots on an interval x scale of Mori’s “human likeness” dimension. By assessing the degree of overlap of probability distributions with means M_1_, M_2_ …, it can predict conditions when psychological *confusion* may occur due to the possible overlap of the distributions of the effects, generated by different stimuli (see Figure 1, left)^5^. We follow the theory of Moore (2012) in assuming the overlap of category distributions as the source of confusion between “human” and “non-human” categories. We propose differing distances between agents—human, robot, or android—on a single *x*-axis. We set as an aim for future research to study cases of different degrees of overlap between distributions of effects from different entities like mechanical robots, humanoid robots, and android robots within certain educational scenarios.

Some accounts of user acceptance of CPSs in terms of the existence of possible higher overlap (due to a slight distortion of the underlying functions of representation) of the distributions of the psychological effects are formulated in recent theories of user interaction with a variety of humanoid or android robots, such as the theory of violation of predictive coding (Saygin et al., 2012; Urgen et al., 2013; Urgen et al., 2018), realism inconsistency theory (MacDorman, and Chattopadhyay, 2016), distortion of categorical perception theory (Moore, 2012), and robot mediation in social control theory (Xu et al., 2023).

The attempts to formulate lawful dependencies in human cognition (based on postulating function distortions) go beyond perception. For example, an account of human memory performance is proposed by Tulving and Wiseman, who derived a function of a “near-independence” relation between the probability to *recognize* a studied item P(Rn) and to *recall* it P(Rc), where P(Rn|Rc) = P(Rn) (1 + δ), or P(Rn|Rc)/P(Rn) = 1 + δ, called the “recognition failure of recallable words” phenomenon (Tulving, and Wiseman, 1975, p.79). This theory argues against the common assumption that the probabilities of recognizing and recalling studied items are highly correlated, i.e., P(Rc) = P(G)P(Rn), where P(G) is the probability of generating a previously studied item and recognizing it as such (Bahrick, 1970).

It is well known that when the conditional probability of an event P(Rn|Rc) with respect to another event P(Rc) equals its probability of occurrence of P(Rn), the two processes are independent (George, 2004; Brereton, 2016) and their plot is diagonal (Figure 1, right)^6^. In the theory proposed by Tulving and Wiseman (1975), this independence relation is slightly violated by a fraction δ, where δ = c[1-P(Rn)], and c is a coefficient in the range (0, 1).

Tulving et al. observed this slight distortion of the independence relation under numerous experimental conditions (Tulving, and Wiseman, 1975; Tulving, and Watkins, 1977), which seems logically linked to the discussed models of the “uncanny valley,” postulating a *slight distortion* of the distributions of the subjective effects, produced by a robot (with a mean M_1_), strongly resembling a human (with a mean M_2_) on the x-axis, leading to a somewhat greater overlap (causing psychological effects, above a certain subjective threshold) than in cases of the effects, produced by completely independent events, as discussed in Moore (2012).

These psychophysical “laws” operate on the abstract representation of the external stimulus in the human mental representation space, which may, or may not, be entirely mutually *congruent* (Osorina and Avanesyan, 2021; Momen et al., 2024), still aiming at creating a meaningful and truthful picture of the objective world. Such a possibility is being supported by studies, mapping linear to nonlinear transformations of psychophysical processes onto neuronal activities in fMRI studies in support of the existence of the “uncanny valley” effect (Rosenthal-Von der Pütten, 2019).

A recent systematic review of the human acceptance of social robots provides evidence of humans having a generally positive attitude toward social robots, hypothesizing the following intriguing possibility, forwarded earlier by Prescott (2017)—to “acknowledge the qualities that mark social robots as not just another technological development but perhaps as an entire *new social group* ^7^ with its own complexity” (Naneva et al., 2020). In the latter work, a new ontological category is being proposed—that of the robots as technological tools, which are perceived as more than just machines, i.e., as entities, possessing some distinctive features of being agents—initiative, autonomy, and reflection. The present paper considers the *psychophysical* nature of the inner, subjective response to this newly emergent complex stimulus—the CPS/robot—with its instantly presented perceptual features of physical, technical, technological, bio-physical, and social appearance.

Until recently, it was possible to assume perceiving a robot as cognitively similar to perceiving variants of non-living things such as puppets, mechanical toys, and avatars. (Dimitrova et al., 2020). However, in recent decades, the CPS/robot category has emerged as a new socio-cultural phenomenon (Prescott, 2017; Naneva et al., 2020; Dimitrova and Wagatsuma, 2015). By existing on the verge of perception (the subjective level of processing the incoming senses from the information) and categorization (the subjective level of retrieving from the memory of knowledge about known and unknown categories of entities), it is possible to expect certain dynamics in the mental representations of robots, tailored to the specific context of usage, as well as some shift toward higher psychological confidence with multifunctional technological devices, than 50 years ago when Masahiro Mori proposed his theory.

As pointed out by Mori, a CPS, or a robot, can be a physical entity with few, or none, biologically inspired features, or it can resemble biological tissue, or even non-living entities like zombies. His proposal is to find the balance between functional *realism* and *aesthetics* in the design of the features of the physical appearance of humanoid agents in order to avoid the “uncanny valley” effect (Mori et al., 2012). To a great extent, this advice was followed while designing the humanoid robot NAO at Aldebaran Robotics, Ltd., commonly used in, for example, interactive scenarios for children (Jackson, and Williams, 2021).

Our approach asserts that the classical and modern psychophysical assumptions describe the regularities of the internal representation of the complex external environment in which the human exists—physical, technological, and social—on different conceptual levels of mental abstraction, reflecting the complexities of the attributes of the objects in the world. Justification for this view was given by Johnson et al. (2002), referring to the classics of psychophysics from a modern perspective: “Fechner seemed to have a clear notion of what had to be done to translate the study of outer psychophysics to the study of inner psychophysics (Fechner 1860, p. 56): “Quantitative dependence of sensation on the [outer] stimulus can eventually be translated into dependence on the [neural activity] that directly underlies sensation—in short, the psychophysical processes—and the measurement of sensation will be changed to one depending on the strength of these processes.” When people coexist with CPSs in various social situations such as at work, home, and hospital, the attributes of the robot are being subjectively processed from many facets, including the psychophysical facet.

## 3 A view on the psychophysical distance between the robot and human agent stimuli

The classical “uncanny valley” function depicts the functional relation between two dimensions—human likeness and affinity (Mori et al., 2012). The horizontal axis x represents the human likeness, whereas the vertical axis y represents the affinity in Mori’s terms. Recent studies split the affinity attribute/feature into familiarity and likability since the original affinity feature is a complex attribute, which is easily understood in Japanese but not possible to translate unambiguously in English. Both familiarity and likability are used to complement or confirm the main assumptions on investigating various factors, which may produce the psychological effect of *uncanny valley* in cases of an encounter with an artificial agent (Rosenthal-Von der Pütten et al., 2019).

In previous studies, we used four types of agents in various roles, such as a toy—a walking robot BigFoot (Nikolov et al., 2022); a zoology teacher—the humanoid robot NAO^8^ (called Roberta) (Dimitrova et al., 2019); and a counseling assistant—the android type of robot SociBot^9^ (called Alice), which was previously used in the studies on psychological reactance to robots by Ghazali et al. (2018) and Ghazali et al. (2020). In our study, the users’ reaction to videos of Alice and Roberta was compared to the reaction to a video of a human actress (by the name of Violina), along six characteristics (plotted on the y axis as a function of x), three positive—sociability, trust, and emotional stability—and three negative—weirdness, threat, and aggression (Dimitrova et al., 2023). Viewers differently assessed the positive and negative features (main effect of factor feature type) of the presented faces —human, android, or robotic (machine-looking). They tend to be cautious in negatively evaluating neutral faces and are inclined to see positive features in these faces to a large extent. This observation deserves further exploration yet suggests certain asymmetry in user attribution of features to the robots, depending on the robots’ intended roles.

**FIGURE 2:**
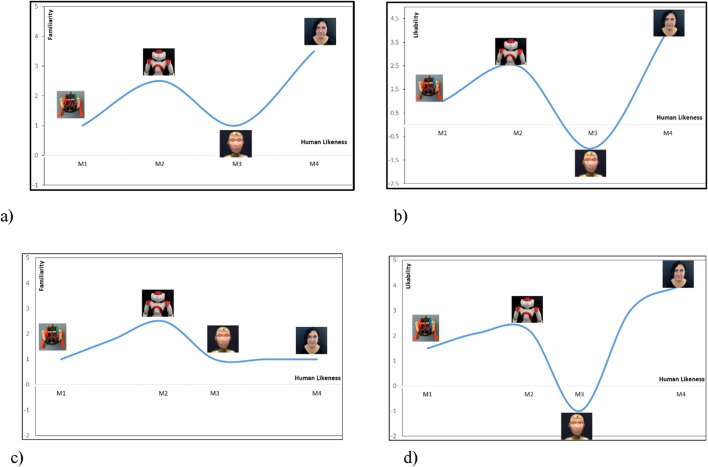
Plots of the expected *uncanny valley* function in four different cases: **(A)** plot of *familiarity* as a function of *human likeness* in the classical case; **(B)** plot of *likability* as a function of *human likeness* in the classical case; **(C)** plot of *familiarity* as a function of human likeness in the proposed case; and **(D)** plot of *likability* as a function of *human likeness* in the proposed case.

Figure 2 plots the hypothetical uncanny valley effect, expected at the encounter of each of the above agents, according to the proposed psychophysical account of robot acceptance by the human. The reactance effect is assumed identical with the uncanny valley effect in terms of its valence but different as *intensity*—manifested on the emotional level in the first case (reactance) and on the visceral level in the second (uncanny valley). Cases (a) and (b) of Figure 2 represent hypothetically the classical view of the *uncanny valley* effect, whereas cases (c) and (d) present the psychophysical view forwarded in the present paper.

The machine-looking toy robot BigFoot is expected to be placed at the left origin of the human likeness dimension in all four cases shown in Figure 2. The robot is the least human-looking (M_1_ on the x axis), least familiar, and, possibly, not *liked* (y axis). The position of the humanoid robot Roberta (M_2_ on the x axis), popular for being designed as cute and likable by children, will possibly be approximately in the same position in all four cases as well—more *human-like* than BigFoot, *familiar* as being quite frequent, and *likable* by design (y axis). The relative positions of the android Alice and the human Violina, however, are expected to differ in the classical and the proposed cases. In the classical case, the uncanny valley function would predict a decrease below the zero line of the *y*-axis with the increase in human-like features of the artificial agent Alice. This decrease below the *y*-axis signifies the affective (negative emotional) reaction to the android, closely resembling perceptually a human agent. Alice is unfamiliar (a) and possibly not much liked because of this (b). In both cases, the human face is expected to be the most familiar and likable (although never seen before).

In case (c), only NAO is familiar, whereas BigFoot, Alice (M_3_ on the x axis), and Violina (M_4_ on the x axis) are never seen before, and the familiarity effect will be similar for all of them (y axis). At the same time, the human likeness is distinctly different in all three robotic cases (x axis). In case (d), it is not quite possible to predict which agent will be most liked (y axis). All faces have neutral expressions, and the classical condition of the expected repulsion by the artificial agent will not hold to the full extent. Still, the characteristic form of the *uncanny valley* function—an increase with Roberta and decrease with Alice—will be observed as a law of perception, holding every time when robots are assessed by their appearance or action.

Most of the existing models of this phenomenon postulate the positions of the agents on the x axis in an *ad hoc* way. Our proposal is to implement the rating procedures for different characteristics on the y axis (trust, likability, familiarity, etc.) plus the indirect scaling procedure of Thurstone to determine the (non-arbitrary) *interval* values with respect to the “human likeness” dimension (x axis)—M_1_, M_2_, M_3_, and M_4_—as the means for the distributions of each category of agents—a mechanical robot, machine-looking humanoid, android, and a human. Moreover, for every role or scenario involving the CPS/robot, and even for each human, interacting with the robot, individual mapping can be performed so that for every user, the level of confidence with any of the robots can be defined. In this way, the proposed psychophysical approach can be used as an overall methodological framework, applicable for the design of CPSs, intended to support users in general and enhance accessibility via CPSs for users with special needs.

Overall, the current analysis is being validated to a certain extent by Mathur, and Reichling (2016). Users were presented with photos of the 80 most popular on-the-web robotic faces, ranging from 0 to max on the *x*-axis of “human likeness.” By fitting the data curves of the robot’s likability, a well-pronounced *uncanny valley* effect was observed. The authors assert that “a formidable android specific problem” exists (p. 22), which we call a lawful dependence of human reaction to CPSs/robots of different perceptual similarities to a human. The machine-looking robot, resembling NAO, obtained the highest score on “likability,” similar to the result that we observed and presented in Dimitrova et al. (2024). Mori’s intuition was supported in terms of a strong link between functionality and aesthetics when designing humanoid robots as a subclass of the class “social CPSs.” Mathur, and Reichling (2016) revealed different effects of the factors likability and trust, which did not predict each other. The authors state the following: “These observations help locate the study of human-android robot interaction squarely in the sphere of human social psychology rather than solely in the traditional disciplines of human factors or human machine interaction” (p. 31). Therefore, if the interaction with social CPSs is governed by the laws of social psychology, the assumption about the newly emergent socially shaped ontology, involving mechanical entities, projecting their “inner states” to the human, gradually acquires more support.

Psychophysics is essential in providing a methodology for the indirect measurements and design of quantitative scales to represent the inner dynamics of perceiving objects in order to avoid negative emotions—a process largely dependent on the individual characteristics of the current user of the CPS. It is also proposed to map professional and other social roles to different types of agents at the stage of interaction design, rather than rely on a unified generalized conceptualization of all possible perceptual varieties of robots and androids engaged in social activities.

## 4 Conclusion

This mini-review considers several psychophysical laws from the point of view of their relevance to model the psychological processes underlying the user acceptance of complex CPSs for social applications. It is demonstrated that the laws of psychophysics are intrinsically related to the theory proposed by Masahiro Mori regarding the function of non-monotonic increase in user affinity for a robot with an increasing degree of its “human likeness.” It is worth investigating human responses to technologically and socially ambiguous stimuli by implementing the psychophysical methodology to reveal the internal dynamics of perceiving the newly designed social agents, performing various social roles. By implementing the indirect scaling methodology useful scenarios for human–robot interaction can be designed, accounting for the individual user-explicit or implicit preferences, accessible and individualized, in order to better adapt to the sensor and learning needs of the users.

## Footnotes

1
https://cordis.europa.eu/project/id/777720

2 The italics is ours.

3 along the *x*-axis (added by us).

4 along the *y*-axis (added by us).

5 Reproduced from Dimitrova et al. (2021b) under Creative Commons Attribution License (CC BY).

6 The simulated function and data are MATLAB generated from the “recognition failure” function of Tulving and Wiseman (1976) for the illustration of the “near-independence” probabilistic relation.

7 Italics is ours.

8
https://www.aldebaran.com/en/nao

9
https://wiki.engineeredarts.co.uk/SociBot
